# Retraction: Cyclophilin A (CypA) Interacts with NF-κB Subunit, p65/RelA, and Contributes to NF-κB Activation Signaling

**DOI:** 10.1371/journal.pone.0339091

**Published:** 2025-12-17

**Authors:** The PLOS One Editors

After this article [1] was published, concerns were raised about Fig 2C.

Specifically:

- In all panels in Fig 2C, several bands appear to be similar to one another. These similarities appear to occur both between bands in different lanes and between bands within the same lane.
- Background patterns appear similar across lanes 9–12, 22–24, and 28–29 and within lanes 14, 25–27 and 30.
- In all panels in Fig 2C, there appear to be multiple vertical and horizontal discontinuities, and multiple areas discontinuous with the adjacent background areas..

The authors were unable to provide the original underlying blots for Fig 2C at editorial request.

In light of the nature and extent of the above concerns which question the reliability and integrity of the results presented in [1], the *PLOS One* Editors retract this article.

SS, KKY, and RHC did not agree with retraction. MG, JBZ, and AH either could not be reached or did not respond directly.
