# Correction: Retraction: Cyclophilin A (CypA) Interacts with NF-κB Subunit, p65/RelA, and Contributes to NF-κB Activation Signaling

**DOI:** 10.1371/journal.pone.0342622

**Published:** 2026-02-11

**Authors:** The PLOS One Editors

## Notice of Republication

The retraction notice [1] for [2] was republished on January 26, 2026, to correct the first author’s statement regarding data availability. The publisher apologizes for the error.

Please download this retraction notice again to view the correct version. The originally published, uncorrected notice and the republished, corrected notice are provided here for reference.

## Supporting information

S1 File Originally published, uncorrected retraction notice. (PDF)

S2 File Republished, corrected retraction notice. (PDF)
