# Supplementary material for: Correction: Retraction: Cyclophilin A (CypA) Interacts with NF-κB Subunit, p65/RelA, and Contributes to NF-κB Activation Signaling
Source: PLoS One. 2026 Feb 11;21(2):e0342622. doi: 10.1371/journal.pone.0342622 (PMC12893544; doi:10.1371/journal.pone.0342622)
Supplement: S2 File — (PDF) [file pone.0342622.s002.pdf]

PLOS One

RETRACTION

Retraction: Cyclophilin A (CypA) Interacts with NF-κB Subunit, p65/RelA, and Contributes to NF-κB Activation Signaling

The *PLOS One* Editors

After this article [1] was published, concerns were raised about Fig 2C.

Specifically:

- In all panels in Fig 2C, several bands appear to be similar to one another. These similarities appear to occur both between bands in different lanes and between bands within the same lane.
- Background patterns appear similar across lanes 9–12, 22–24, and 28–29 and within lanes 14, 25–27 and 30.
- In all panels in Fig 2C, there appear to be multiple vertical and horizontal discontinuities, and multiple areas discontinuous with the adjacent background areas..

The authors were unable to provide the original underlying blots for Fig 2C at editorial request.

In light of the nature and extent of the above concerns which question the reliability and integrity of the results presented in [1], the *PLOS One* Editors retract this article.

SS, KKY, and RHC did not agree with retraction. MG, JBZ, and AH either could not be reached or did not respond directly.

Reference

1. Sun S, Guo M, Zhang JB, Ha A, Yokoyama KK, Chiu RH. RETRACTED: Cyclophilin A (CypA) interacts with NF-κB subunit, p65/RelA, and contributes to NF-κB activation signaling. PLoS One. 2014;9(8):e96211. https://doi.org/10.1371/journal.pone.0096211 PMID: 25119989

Check for updates

OPEN ACCESS

Citation: The *PLOS One* Editors (2025) Retraction: Cyclophilin A (CypA) Interacts with NF-κB Subunit, p65/RelA, and Contributes to NF-κB Activation Signaling. PLoS One 20(12): e0339091. https://doi.org/10.1371/journal.pone.0339091

Published: December 17, 2025

Copyright: © 2025 The *PLOS One* Editors. This is an open access article distributed under the terms of the Creative Commons Attribution License, which permits unrestricted use, distribution, and reproduction in any medium, provided the original author and source are credited.
